# Identification of D Modification Sites by Integrating Heterogeneous Features in *Saccharomyces cerevisiae*

**DOI:** 10.3390/molecules24030380

**Published:** 2019-01-22

**Authors:** Pengmian Feng, Zhaochun Xu, Hui Yang, Hao Lv, Hui Ding, Li Liu

**Affiliations:** 1 Innovative Institute of Chinese Medicine and Pharmacy, Chengdu University of Traditional Chinese Medicine, Chengdu 611730, China; 2 Key Laboratory for Neuro-Information of Ministry of Education, School of Life Science and Technology, Center for Informational Biology, University of Electronic Science and Technology of China, Chengdu 610054, China; jdzxuzhaochun@163.com (Z.X.); huiyang0325@163.com (H.Y.); 13208188368@163.com (H.L.); hding@uestc.edu.cn (H.D.); 3 Computer Department, Jingdezhen Ceramic Institute, Jingdezhen 333403, China; 4 Laboratory of Theoretical Biophysics, School of Physical Science and Technology, Inner Mongolia University, Hohhot 010021, China; 5 School of Public Health, North China University of Science and Technology, Tangshan 063000, China

**Keywords:** dihydrouridine, nucleotide physicochemical property, pseudo dinucleotide composition, RNA secondary structure, ensemble classifier

## Abstract

As an abundant post-transcriptional modification, dihydrouridine (D) has been found in transfer RNA (tRNA) from bacteria, eukaryotes, and archaea. Nonetheless, knowledge of the exact biochemical roles of dihydrouridine in mediating tRNA function is still limited. Accurate identification of the position of D sites is essential for understanding their functions. Therefore, it is desirable to develop novel methods to identify D sites. In this study, an ensemble classifier was proposed for the detection of D modification sites in the *Saccharomyces cerevisiae* transcriptome by using heterogeneous features. The jackknife test results demonstrate that the proposed predictor is promising for the identification of D modification sites. It is anticipated that the proposed method can be widely used for identifying D modification sites in tRNA.

## 1. Introduction

To date, more than 100 kinds of post-transcriptional modifications have been identified in transfer RNAs (tRNAs). It has been demonstrated that these modifications are involved in all core aspects of tRNA function [1]. Among them, dihydrouridine (D) is a prevalent tRNA modification, which has been found in the three domains of life [2].

The D modification is formed by a dihydrouridine synthase [3]. Unlike uridine (U), the ring of D is not aromatic, which precludes its interactions with other bases in tRNA by stacking interactions [4,5]. By destabilizing the tRNA structure, D can enhance the conformational flexibility of tRNA [6]. Therefore, it is concluded that the flexibility and even the folding of tRNA could be affected by D modification [4,7]. 

Recent studies have also shown that tRNA lacking D degrades significantly faster, suggesting that D modification can protect tRNAs from degradation [1,8]. Despite the abundant occurrence of D modification, our knowledge about its roles in mediating tRNA biological functions is still limited. Therefore, it is urgent to develop novel methods to describe the distribution of D modification sites. Since it is cost ineffective and labor intensive to detect D modification sites by using experimental techniques, it is necessary to develop theoretical methods for the detection of D modification.

Therefore, in the present study, an ensemble classifier was proposed for the detection of D modification sites in the *Saccharomyces cerevisiae* transcriptome, in which the nucleotide physicochemical property, pseudo dinucleotide composition, and secondary structure component were employed to train the basic predictors, respectively. In the jackknife test, the ensemble classifier obtained an accuracy of 83.09% for identifying D modification sites. This result demonstrated the superiority of the proposed method for identifying D modification sites in the *S. cerevisiae* transcriptome.

## 2. Results

### 2.1. Performances of Different Features

In order to demonstrate the effectiveness of the different kinds of features for identifying D sites, we first built support vector machine (SVM) predictors based on each kind of sequence encoding schemes (i.e., nucleotide physicochemical property, pseudo dinucleotide composition, or secondary structure component). Their jackknife test results for identifying D sites in the *S. cerevisiae* transcriptome are reported in Table 1. Although the nucleotide-physicochemical-property-based predictor (NPCP-SVM) obtained the highest accuracy (Acc) for identifying D sites, its sensitivity (Sn) was only 67.65%, indicating that it still could not accurately identify the real D sites. For the predictors based on pseudo dinucleotide composition and secondary structure component (namely PseDNC-SVM and SSC-SVM), their accuracies (Acc) were only 75.74% and 72.79% with the atthews correlation coefficients (MCC) of 0.5 and 0.45, respectively. Taken together, these results indicate that the performances of the aforementioned three predictors were not fully satisfactory. Therefore, there is still scope to improve the performance for identifying D sites.

### 2.2. Improving Predictive Performance Using Ensemble Learning

**Figure 1:**
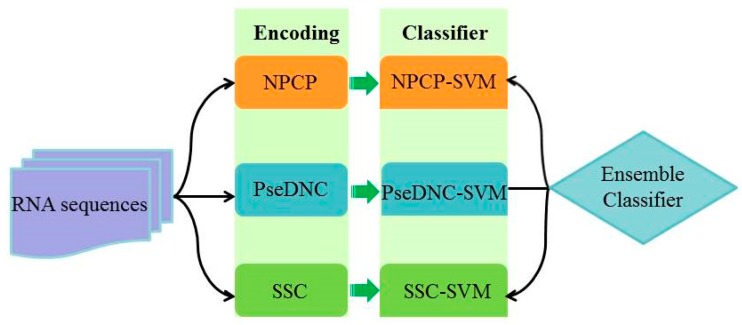
The flaw chart of the ensemble classifiers. NPCP-SVM stands for nucleotide-physicochemical-property-based predictor; PseDNC-SVM stands for pseudo-dinucleotide-composition-based predictor; SSC-SVM stands for secondary-structure-based predictor.

Several recent works have demonstrated that the ensemble learning scheme can improve the performance of predictors [9,10,11,12,13]. In order to improve the performance of identifying D sites, we constructed an ensemble predictor based on SVM by using different kinds of features. Therefore, three basic SVM-based predictors were built by using nucleotide physicochemical property, pseudo dinucleotide composition, and secondary structure component, respectively. Figure 1 shows the prediction process with the ensemble classifier. The three predictors were integrated as an ensemble predictor via a voting strategy (see Materials and Methods). By combining the results of the three predictors together, a sequence in the benchmark dataset was predicted as a D-site-containing sequence if its prediction probabilities yielded by more than two predictors were all greater than 0.5.

The jackknife test results of the ensemble predictor for identifying D sites in *S. cerevisiae* transcriptome are also listed in Table 1. It was found that the sensitivity of the ensemble predictor was improved to 76.47%. Although its specificity and accuracy was a little lower than NPCP-SVM, the MCC of the ensemble predictor was 0.62, which was higher than that of any single SVM-based predictor, indicating the ensemble predictor was much more stable than NPCP-SVM, PseDNC-SVM, and SSC-SVM for the detection of D modification sites.

## 3. Materials and Methods

### 3.1. Benchmark Dataset

The original 208 positive samples (D-site-containing sequences) were fetched from the RMBase database [14]. All of these sequences in RMBase were 41 nt long with the D site in the center. Preliminary tests indicated that the best prediction results were achieved when the sequence was 41 nt long. In order to avoid redundancy, sequences with more than 80% sequence similarity were removed using the CD-HIT program [15]. Accordingly, we obtained 68 D-site-containing sequences from the *S. cerevisiae* transcriptome.

Negative samples were obtained by selecting 41-nt-long sequences that satisfied the following rules: (1) uridine is the center of the sequence, and (2) no dihydrouridine modification of the centered uridine has been identified experimentally. Accordingly, we could obtain a huge number of negative samples, from which we randomly picked 68 samples to form the negative subset for the purpose of using a balance benchmark dataset to train the model. In summary, our benchmark dataset comprised 68 D-site-containing sequences and 68 false D-site-containing sequences from the *S. cerevisiae* transcriptome, which is available at https://github.com/chenweiimu/D-Pred.

### 3.2. Sequence Encoding Scheme

#### 3.2.1. Nucleotide Physicochemical Property (NPCP)

Adenosine (A), cytosine (C), guanine (G), and uridine (U) have different chemical properties [16,17]. In terms of ring structures, A and G are purines containing two rings, whereas C and U are pyrimidines containing one ring. When forming secondary structures, C and G form strong hydrogen bonds, whereas A and U form weak hydrogen bonds. In terms of amino/keto bases, A and C belong to the amino group, while G and U belong to the keto group [16,17].

In order to encode RNA sequences using these properties, the (*x*, *y*, *z*) coordinates were used to describe the chemical properties of the four nucleotides, and a value of 0 or 1 was assigned to (*x*, *y*, *z*), respectively. If *x*, *y*, and *z* coordinates stand for the ring structure, the hydrogen bond, and the amino/keto bases, A, C, G, and U can be represented by (1, 1, 1), (0, 0, 1), (1, 0, 0), and (0, 1, 0), respectively.

Accordingly, by using nucleotide chemical properties, each sequence could be encoded by a 123 (3 × 41)-dimensional vector, as given bellow:

(1) $$R_{1}={\left[\begin{array}{ccccccc} \varepsilon_{1} & \varepsilon_{2} & \varepsilon_{3} & \cdots & \varepsilon_{i} & \cdots & \varepsilon_{123} \end{array}\right]}^{T}$$

where *ε_i_* indicates the abovementioned nucleotide chemical properties, and its value is 0 or 1.

#### 3.2.2. Pseudo Dinucleotide Composition

The pseudo *k*-tuple nucleotide composition (PseKNC), proposed by Chen et al. [18,19], has been successfully and widely applied in computational genomics [20,21,22]. PseKNC not only includes local sequence order information but also the global sequence pattern [23]. In the current study, the pseudo dinucleotide composition (PseDNC) was used to encode the RNA sequences and is defined as follows [18,19]:

(2) $$R={\left[\begin{array}{ccccccc} d_{1} & d_{2} & \cdots & d_{16} & d_{16+1} & \cdots & d_{16+\lambda } \end{array}\right]}^{T}$$

where

(3) $$d_{u}=\{\begin{array}{cc} \frac{f_{u}}{\sum_{i=1}^{16}f_{i}+w\sum_{j=1}^{\lambda }\theta_{j}} & \left(1\leq u\leq 16\right)\\ \frac{w\theta_{u-16}}{\sum_{i=1}^{16}f_{i}+w\sum_{j=1}^{\lambda }\theta_{j}} & \left(16<u\leq 16+\lambda \right) \end{array}.$$

In Equation (3), $f_{u}$ (u=1,2, ⋯, 16) is the normalized occurrence frequency of the u-th nonoverlapping dinucleotide in the RNA sequence, and

(4) $$\begin{array}{cc} \theta_{j}=\frac{1}{L-j-1}\sum_{i=1}^{L-j-1}C_{i,\;i+j} & \left(j=1,2,\;\cdots ,\;\lambda ;\;\lambda <L\right) \end{array}$$

where $\theta_{j}$ is the *j*-tier correlation factor that reflects the sequence order correlation between all the *j*-th most contiguous dinucleotides. The coupling factor $C_{i,\;i+j}$ is defined as

(5) $$C_{i,\;i+j}=\frac{1}{\mu }\overset{\mu }{\underset{g=1}{\sum }}\;{\left[P_{g}\left(D_{i}\right)-P_{g}\left(D_{i+j}\right)\right]}^{2}$$

where *μ* is the number of RNA physicochemical properties considered, $P_{g}\left(D_{i}\right)$ is the normalized numerical value of the *g*-th (*g* = 1, 2, 3, …, *μ*) RNA local structural property for the dinucleotide R*_i_*R*_i_*_+1_ at position *i*, and $P_{g}\left(D_{i+j}\right)$ is the corresponding value for the dinucleotide R*_i_*_+*j*_R*_i_*_+*j*+1_ at position *i* + *j*.

Inspired by a recent study [24], the three RNA physicochemical properties, namely, enthalpy [25], entropy [25], and free energy [26], were used to define PseDNC. Thus, in Equation (4), *μ* is equal to 3. The normalized numerical values of the three physicochemical properties of the 16 different RNA dinucleotides were obtained from our previous work [24].

The two parameters *w* and *λ* were optimized in the following ranges [0, 1] and [1, 10] with steps of 0.1 and 1, respectively. In the current work, the optimal values for *w* and *λ* were 0.5 and 4, respectively. Hence, the RNA sequence can be formulated by a (16 + 4) = 20-dimensional vector as given below:

(6) $$R_{2}={\left[\begin{array}{ccccccc} d_{1} & d_{2} & \cdots & d_{16} & d_{17} & \cdots & d_{20} \end{array}\right]}^{T}$$

#### 3.2.3. Secondary Structure Component (SSC)

Considering the fact that RNA modification is affected by its structures [27], the RNA sequences were also encoded using the RNA secondary structures. By using the RNAfold tool (version 2.1.9) in ViennaRNA package with default parameters [28], we obtained the secondary structure status at each position, which was represented by brackets (“(” or “)”) indicating paired nucleotides and by dots (“.”) indicating unpaired nucleotides. In the current study, we did not distinguish “(” and “)” and used “(” for both situations. For a given trinucleotide, there were eight (2^3^) possible structure statuses (i.e., “(((”, “((.”, “(..”, “(.(”, “.((”, “.(.”, “..(”, and “…”). If the first nucleotide in the trinucleotide was further considered, there would be 32 (4 × 8) possible sequence-structure modes, which were denoted as “A-(((”, “A-((.”, “A-(..”, …, and “U-…”. Therefore, a given sequence could be represented by using the following sequence-structure:

(7) $$R_{3}={\left[f_{(((}^{A},\;f_{((.}^{A},\;f_{(..}^{A},\ldots ,\;f_{\ldots }^{A},\;f_{(((}^{C},\ldots ,f_{\ldots }^{U}\right]}^{T}.$$

The elements in the vector of R_3_ indicate the frequency of the 32 sequence-structure modes.

### 3.3. Support Vector Machine

SVM is a well-known machine learning method for pattern recognition and has been widely used in bioinformatics [29,30,31,32,33,34,35]. In the current study, the LibSVM package 3.18 (http://www.csie.ntu.edu.tw/~cjlin/libsvm/) was used to perform SVM. Due to its effectiveness and speed in training process, the radial basis kernel function (RBF) of SVM was often used to find the classification hyperplane. The regularization parameter *C* and kernel parameter *γ* of the SVM operation engine was optimized in the ranges of [2^−5^, 2^15^] and [2^−15^, 2^−5^] with steps of 2 and 2^−1^, respectively. The prediction was made according to the probability score yielded from SVM. If its probability score was greater than 0.5, a uridine would be predicted as a D site, otherwise, a non-D-site.

### 3.4. Ensemble Classifiers

By using the NPCP, PseKNC, and SSC features, three basic classifiers were built, which voted for the final result according to the following rule [9]:

(8) $$\begin{array}{cc} V_{i}=\overset{3}{\underset{k=1}{\sum }}f\left(pre\left(C_{k}\right),Class_{i}\right) & \left(i=1,\;2\right) \end{array}$$

where *V_i_* is the voting score for the sequence belonging to the Class*_i_*. *f*(*pre*(*C_k_*),Class*_i_*) is defined as

(9) $$f\left(pre\left(C_{k}\right),Class_{i}\right)=\{\begin{array}{cc} 1 & if\;pre\left(C_{k}\right)\in Class_{i}\\ 0 & if\;pre\left(C_{k}\right)\notin Class_{i} \end{array}\;\;\left(i=1,\;2;k=1,\;2,\;3\right).$$

The final prediction is determined by

(10) $$\begin{array}{cc} Sgn\left(i\right)=\arg\max_{i}\left\{V_{i}\right\} & \left(i=1,\;2\right) \end{array}.$$

Sgn(*i*) is the argument that maximizes the voting score *V_i_*.

### 3.5. Performance Evaluation

The performance of the method were evaluated by using sensitivity (Sn), specificity (Sp), accuracy (Acc), and the Matthews correlation coefficient (MCC), as given below [36,37,38,39,40]:

(11) $$\left\{ \begin{array}{ll} \mathrm{Sn}=1-\frac{N_{-}^{+}}{N^{+}} & 0\;\leq \mathrm{Sn}\leq 1\\ \mathrm{Sp}=1-\frac{N_{+}^{-}}{N^{-}} & 0\leq \mathrm{Sp}\leq 1\\ \mathrm{Acc}=1-\frac{N_{-}^{+}+N_{+}^{-}}{N^{+}+N^{-}} & 0\leq \mathrm{Acc}\leq 1\\ \mathrm{MCC}=\frac{1-\left(\frac{N_{-}^{+}}{N^{+}}+\frac{N_{+}^{-}}{N^{-}}\right)}{\sqrt{\left(1+\frac{N_{+}^{-}-N_{-}^{+}}{N^{+}}\right)\;\left(1+\frac{N_{-}^{+}-N_{+}^{-}}{N^{-}}\right)}} & -1\leq \mathrm{MCC}\leq 1 \end{array}\right.$$

where $N^{+}$ represents the total number of D-site-containing sequences, while $N_{-}^{+}$ is the number of D-site-containing sequences incorrectly predicted to be of false D-site-containing sequences. $N^{-}$ is the total number of false D-site-containing sequences, while $N_{+}^{-}$ the number of the false D-site-containing sequences incorrectly predicted to be of D-site-containing sequences.

### 3.6. Jackknife Cross-Validation

Among the three methods (i.e., independent dataset test, K-fold cross-validation test, and jackknife cross-validation), the jackknife cross-validation is deemed to be the least arbitrary, as demonstrated by in a recent review paper [41]. In the jackknife cross-validation, each sample in the training dataset is in turn singled out as an independent test sample and all the rule parameters are calculated without including the one being identified [42,43,44,45,46]. Accordingly, jackknife cross-validation was also used to examine the performance of the method proposed in the current study.

## 4. Conclusions

In this study, by integrating heterogeneous sequence-based features, a SVM-based ensemble classifier was proposed to identify D modification sites in the *S. cerevisiae* transcriptome. In this predictor, not only was the local and global sequence information included by encoding RNA sequences using PseDNC, but the nucleotide chemical properties and structures were also considered by representing RNA sequences using nucleotide physicochemical properties and predicted RNA secondary structures. The jackknife test results demonstrate that the proposed predictor is promising for the identification of D modification sites. It is anticipated that the proposed method will become an essential computational tool for identifying D modification sites in tRNA. 

However, the proposed method has two flaws. The limited number of experimentally verified D modification data hindered us from extracting effective features to describe the D modification sites containing sequences. The other shortcoming is that the present method directly uses the entirety of the features, which may reduce the generalization capacity of the model and increase the computational time. Therefore, in future work, we shall make efforts to collect more D modification data and also employ the feature selection method to winnow out the optimal features.

## Figures and Tables

**Table 1:** Performances of different methods for identifying dihydrouridine (D) sites.

Methods	Sn (%)	Sp (%)	Acc (%)	MCC
NPCP-SVM	67.65	100	83.82	0.59
PseDNC-SVM	73.53	77.94	75.74	0.50
SSC-SVM	70.59	75.00	72.79	0.45
Ensemble SVM	76.47	89.71	83.09	0.62
